# CO_2_ Adsorption over 3*d* Transition-Metal Nanoclusters Supported on Pyridinic N_3_-Doped Graphene: A DFT Investigation

**DOI:** 10.3390/ma15176136

**Published:** 2022-09-04

**Authors:** Fernando Montejo-Alvaro, Jesus A. Martínez-Espinosa, Hugo Rojas-Chávez, Diana C. Navarro-Ibarra, Heriberto Cruz-Martínez, Dora I. Medina

**Affiliations:** 1 Tecnológico Nacional de México, Instituto Tecnológico del Valle de Etla, Abasolo S/N, Barrio del Agua Buena, Santiago Suchilquitongo, Oaxaca 68230, Mexico; 2 Tecnologico de Monterrey, School of Engineering and Sciences, Atizapán de Zaragoza, Estado de México 52926, Mexico; 3 Tecnológico Nacional de México, Instituto Tecnológico de Tláhuac II, Camino Real 625, Col. Jardines del Llano, San Juan Ixtayopan, Alcaldía Tláhuac, Ciudad de México 13550, Mexico

**Keywords:** CO_2_ adsorption and activation, HUMO-LUMO gap, charge transfer, stability

## Abstract

CO_2_ adsorption on bare 3*d* transition-metal nanoclusters and 3*d* transition-metal nanoclusters supported on pyridinic N_3_-doped graphene (PNG) was investigated by employing the density functional theory. First, the interaction of Co_13_ and Cu_13_ with PNG was analyzed by spin densities, interaction energies, charge transfers, and HUMO-LUMO gaps. According to the interaction energies, the Co_13_ nanocluster was adsorbed more efficiently than Cu_13_ on the PNG. The charge transfer indicated that the Co_13_ nanocluster donated more charges to the PNG nanoflake than the Cu_13_ nanocluster. The HUMO-LUMO gap calculations showed that the PNG improved the chemical reactivity of both Co_13_ and Cu_13_ nanoclusters. When the CO_2_ was adsorbed on the bare 3*d* transition-metal nanoclusters and 3*d* transition-metal nanoclusters supported on the PNG, it experienced a bond elongation and angle bending in both systems. In addition, the charge transfer from the nanoclusters to the CO_2_ molecule was observed. This study proved that Co_13_/PNG and Cu_13_/PNG composites are adequate candidates for CO_2_ adsorption and activation.

## 1. Introduction

Due to environmental changes such as rising global temperatures, sea levels, and melting of the polar ice caps, concerns are accumulating around the presence of CO_2_ in the atmosphere [1]. Furthermore, population growth and rapid industrialization have escalated the release of CO_2_ into the atmosphere [2]. In addition, energy requirements for human activities (anthropogenic) have contributed substantially to net CO_2_ emissions [3]. In fact, anthropogenic CO_2_ emissions have dramatically increased from 27 Gt in 1970 to 49 Gt in 2010 [4]. As a result, many governments have signed the Kyoto Protocol of the United Nations Framework Convention on Climate Change as a precautionary measure to mitigate climate change [5].

Additionally, several technological processes have been implemented to curb CO_2_ accumulation by implementing techniques such as the CO_2_ capturing and sequestration process [6], CO_2_ capturing and utilization processes [7], and CO_2_ conversion [8,9]. Yet, the enormous dissociation energy of CO_2_ resulting from its molecular stability limits the feasibility of the latter process [10]. Moreover, challenges in developing suitable catalysts in terms of efficiency and selectivity still stand unresolved [11]. That notwithstanding, strides in this area continually seek to find more efficient catalysts that diminish process energy requirements and show high selectivity and stability to CO_2_ reduction reactions.

In this regard, a wide variety of catalysts have been studied: primarily, current studies have been centered on nanoclusters, since they exhibit superior catalytic activity with respect to bulk materials handling, enabled by their specific surface area, electronic, optical, magnetic, and mechanical properties, which are strongly associated with their shape, size, and composition [12]. Thus, 3*d* transition-metal nanoclusters have gained considerable attention since they have demonstrated remarkable performance in CO_2_ reduction. For instance, the removal and conversion of CO_2_ into liquid fuels using amorphous Cu nanoparticles showed an excellent catalytic activity and selectivity [13]. In another study, Li and colleagues studied the CO_2_ reduction over Ni-based electrocatalysts with sizes ranging from a few atoms to over 100 nm. Their results imply that CO_2_ reduction and selectivity performance vary according to the size of Ni nanoparticles [14]. Furthermore, another study used Co nanoparticles ranging from 10 to 50 nm prepared for CO_2_ methanation and ethanol reforming, demonstrating the nanoparticles’ significant activity in the process [15].

Though the catalytic activities of nanoclusters are acclaimed, they tend to agglomerate, resulting in a decline in activity and stability [16,17,18]. Therefore, addressing this issue requires the use of high-surface-area nanomaterials to anchor nanoparticles to promote stability and activation. One of the most eminent nanomaterials in this respect is graphene which exhibits high mechanical, thermal, and electrical properties [19,20,21]. Nonetheless, graphene often necessitates modifications such as doping, functionalization, or the presence of defects on its surface. These adjustments are intended to overcome the low chemical activity of graphene [21,22]. Among the different modifications made to graphene, the pyridinic N_3_-doped graphene (PNG) has acquired great importance since it substantially modifies the structural and electronic properties of the pristine graphene. Furthermore, studies based on density functional theory (DFT) have proved that the stability and reactivity of metal nanoclusters were enhanced using PNG layers as support [23,24,25].

Moreover, using 3*d* transition-metal nanoclusters supported on PNG for CO_2_ reduction has been studied experimentally. For example, Dongare and collaborators investigated electrochemical CO_2_ reduction using N-doped graphene-supported Cu nanoparticles. The presence of pyridinic, pyrrolic, and graphitic N were confirmed using X-ray photoelectron spectroscopy (XPS). Furthermore, the high PNG content signified good selectivity toward CO_2_ reduction. In the end, the N-doped graphene/Cu nanoparticles composite enhanced the activity and selectivity in CO_2_ reduction [26]. Likewise, the incorporation of MnO nanoparticles into an N-doped graphene aerogel for CO_2_-to-CO electrochemical reduction was studied. According to XPS spectra, it was found that the primary N species was the pyridinic type. Their results suggest that CO_2_-to-CO electrochemical reduction was enhanced by the synergistic effect of the MnO nanoparticles and the N-doped aerogels [27]. Thus, these studies emphasize the central role of N-doped graphene in CO_2_ reduction. However, there are no theoretical studies that analyze the effect of the PNG support on the stability and catalytic activity of 3*d* transition-metal clusters toward CO_2_ at the molecular level. Therefore, in this work, CO_2_ adsorption and activation on bare 3*d* transition-metal nanoclusters and 3*d* transition-metal supported nanoclusters were investigated using DFT calculations, since their adsorption and activation on surfaces are key steps in the CO_2_ conversion reaction [28]. To analyze the CO_2_ interaction on both bare nanoclusters and nanoclusters supported on PNG, the CO_2_ adsorption energy, CO_2_ bond elongation, CO_2_ bending angle, and charge transfer are calculated since these are indicators of effective CO_2_ dissociation [29,30,31,32].

## 2. Materials and Methods

To obtain the most stable CO_2_ adsorption on both the bare nanoclusters and nanoclusters supported on PNG graphene, nine initial structures were considered. All initial structures were optimized using the auxiliary density functional theory (ADFT) method implemented in the deMon2k 4.3.8 software [33]. The ADFT is a reliable and efficient alternative to the conventional DFT approach that allows calculations of large complex systems with less computational effort. The revised Perdew–Burke–Ernzerhof (revPBE) functional was used as the exchange–correlation functional [34]. For the Co, H, C, O, and N atoms, a double-zeta valence plus polarization basis set optimized for generalized gradient approximation (GGA) functionals (DZVP-GGA) was employed [35], and a triple-zeta valence plus polarization basis set optimized for GGA functionals (TZVP-GGA) was used for the Cu atoms [35]. The most stable structures obtained with the ADFT method were reoptimized with the conventional DFT method using the Orca 5.0 software [36]. All DFT calculations were performed using revPBE [34]. The Ahlrichs basis set def2-SVP was used for the C, N, O, and H atoms, and def2-TZVP was used for the Co and Cu atoms [37]. The energy change = 5 × 10^−6^ Eh, max. gradient = 3 × 10^−4^ Eh/Bohr, RMS gradient = 1 × 10^−4^ Eh/Bohr, max. displacement = 4 × 10^−3^ Bohr, and RMS displacement = 2 × 10^−3^ Bohr were the convergence criteria used for geometry optimization.

**Figure 1:**
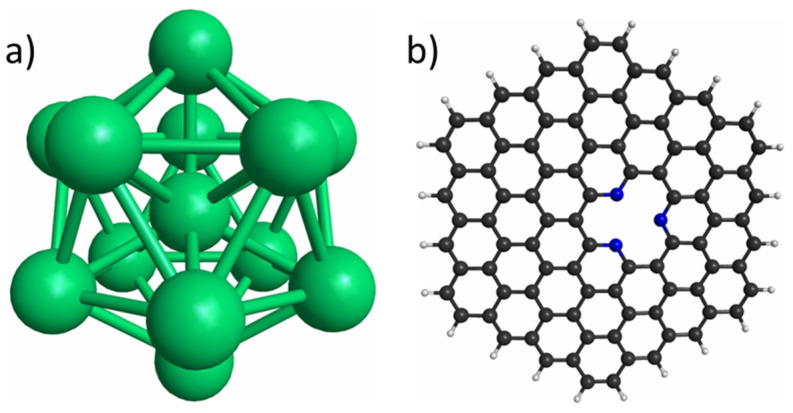
(**a**) Icosahedral metal nanoclusters with 13 atoms; (**b**) Pyridinic N_3_-doped graphene. Green, black, white, and blue spheres represent metal (Co or Cu), C, H, and N atoms, respectively.

The icosahedral Co_13_ and Cu_13_ nanoclusters (Figure 1a) and the graphene model (Figure 1b) used in this work are reported in Figure 1.

The CO_2_ adsorption energies (E_ads_) on isolated nanoclusters and nanoclusters supported on PNG were calculated using the basis set superposition error (BSSE) [38]. Moreover, the atom-pairwise (atom-triplewise) dispersion (D3) correction was used for the RevPBE functional using optimized parameters by Grimme et al. [39]. Finally, to analyze the molecular interactions of the Co_13_ and Cu_13_ nanoclusters supported on PNG and the CO_2_ adsorption over bare nanoclusters and nanoclusters supported on PNG, the quantum theory of atoms in molecules implemented in the Multiwfn program was used [40].

## 3. Results

### 3.1. Properties of Co_13_, Cu_13_, Co_13_/PNG, and Cu_13_/PNG

**Figure 2:**
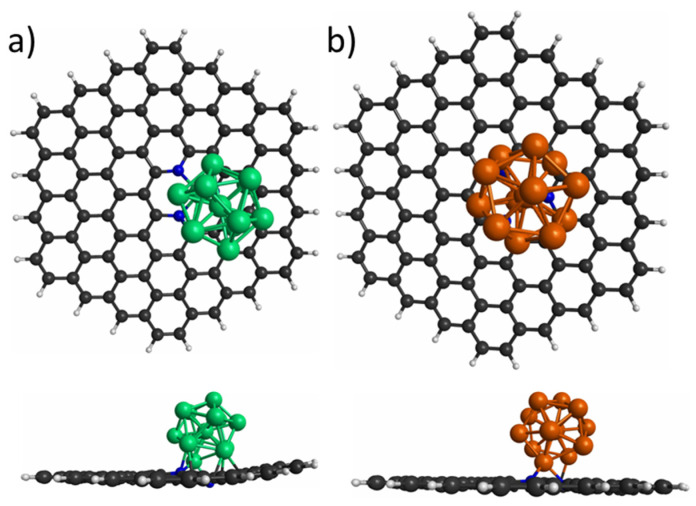
Top and side views of the most stable interaction of (**a**) Co_13_ and (**b**) Cu_13_ nanocluster deposited on pyridinic N_3_-doped graphene. Green, orange, black, white, and blue spheres represent Co, Cu, C, H, and N atoms, respectively.

First, comparison of the spin multiplicities of bare nanoclusters and nanoclusters supported on the PNG was made. The Co_13_ and Cu_13_ nanoclusters possess spin multiplicity values of 32 and 6, respectively, which agree with previous results reported for the Co_13_ [41] and Cu_13_ [42] nanoclusters. The optimized Co_13_ and Cu_13_ nanoclusters supported on PNG are illustrated in Figure 2. When the Co_13_ and Cu_13_ nanoclusters are supported on PNG, a decrement in spin multiplicity was observed in the supported nanoclusters compared to the bare nanoclusters. The Co_13_/PNG composite ended with a spin multiplicity value of 27, whereas the Cu_13_/PNG composite was singlet. The spin multiplicity diminution observed in both systems is ascribed to the stabilizing effect of the PNG. This is also consistent with data reported in literature. For instance, for the Pd- and Pt-based nanoclusters supported on the PNG nanoflake, the latter lead to a magnetic-moment decrement in most of the systems; such behavior was associated with the charge transferred from the nanoclusters to the PNG nanoflake [23,24].

**Figure 3:**
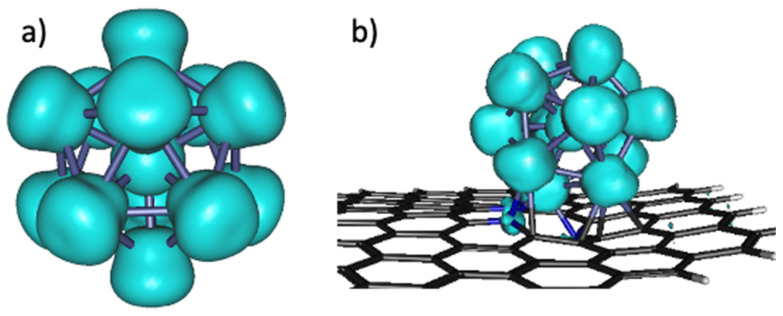
Spin density distributions of the systems: (**a**) Co_13_ nanocluster and (**b**) Co_13_/PNG composite.

Spin densities, interaction energies (E_int_), and charge transfers were calculated to better understand the interaction between the nanoclusters and PNG support. In Figure 3 the spin density of the Co_13_ nanocluster supported on the PNG is illustrated, since it is an open-shell system, and the spin density of the Co_13_ nanocluster is also computed for comparison. As can be seen in Figure 3a, for the Co nanocluster, there is a homogeneous spin density distribution over the Co atoms. When the Co nanocluster is supported on the PNG, the spin density is distributed mostly on the Co atoms and little on the N atoms (Figure 3b).

The E_int_ calculated for the Co_13_ and Cu_13_ nanoclusters supported on PNG are −5.69 eV and −4.72 eV, respectively. It should be noted that both values are substantially higher than those reported in previous findings for the Co_13_ [43,44] and Cu_13_ [45,46] nanoclusters supported on pristine graphene. Therefore, PNG has a more substantial stabilizing effect over the Co_13_ and Cu_13_ nanoclusters compared to pristine graphene. The interaction between the Co_13_ and Cu_13_ nanoclusters with PNG was further investigated by Bader charge transfer. The charge transfer between the Co_13_ and Cu_13_ nanoclusters and the PNG is reported in Table 1. The results suggest that both nanoclusters yielded charge to the PNG since both ended with a total positive charge. The large electronegativity of N and the electronegativity difference of Co and Cu atoms are the principal driving force for the charge transfer. Furthermore, the highest occupied molecular orbital (HOMO) and lowest unoccupied molecular orbital (LUMO) gap was computed for the bare Co_13_ and Cu_13_ nanoclusters and the Co_13_/PNG and Cu_13_/PNG composites, Table 1. The Co_13_ and Cu_13_ nanoclusters show HOMO-LUMO gaps of 0.52 and 0.67 eV, respectively, which imply that the Co_13_ can be more reactive than the Cu_13_ nanocluster as it has been documented that a low HOMO-LUMO gap is associated with high chemical reactivity [45,47,48]. The HOMO-LUMO gaps of the Co_13_/PNG (0.26 eV) and Cu_13_/PNG (0.21 eV) were lower than that for the bare nanoclusters. Therefore, it is assumed that the presence of the PNG increased the chemical reactivity of the nanoclusters.

### 3.2. CO_2_ Adsorption on Co_13_ and Cu_13_ Nanoclusters

**Figure 4:**
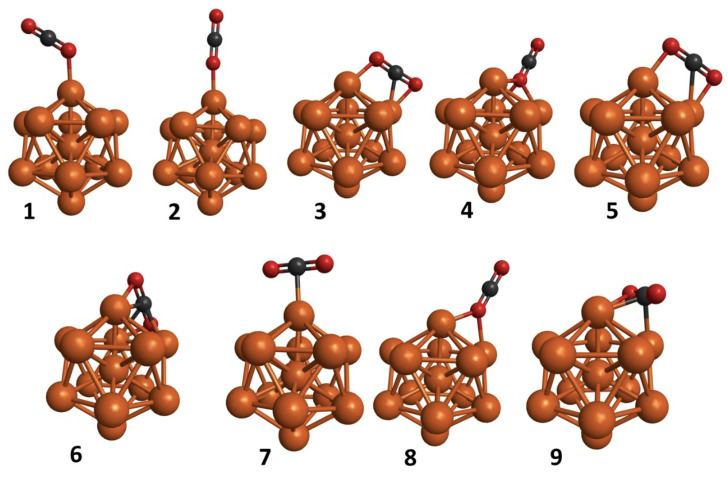
CO_2_ adsorption modes on nanoclusters.

Several modes of CO_2_ adsorption on both the bare nanoclusters and nanoclusters supported on PNG were investigated. First, the CO_2_ molecule was placed over different facets of the Co_13_ and Cu_13_ nanoclusters to identify the most optimal adsorption modes. Due to the high symmetry of the icosahedral Co_13_ and Cu_13_ nanoclusters, only nine adsorption modes were considered (Figure 4). Specific parameters were considered to evaluate the CO_2_ adsorption on the Co_13_ and Cu_13_ nanoclusters, such as the E_ads_, the average bond length, the bending angle of the CO_2_ molecule, and the charge transfer from the nanoclusters to the CO_2_ molecule. These parameters were calculated over the most stable structures of CO_2_ adsorption on nanoclusters and nanoclusters supported on PNG.

**Figure 5:**
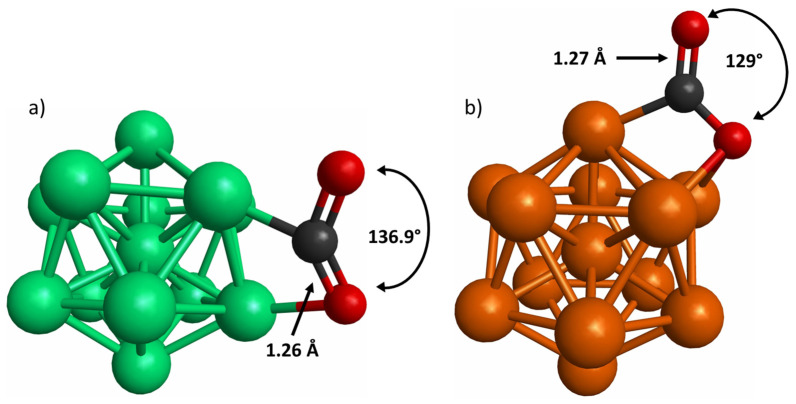
CO_2_ adsorbed on (**a**) Co_13_ and (**b**) Cu_13_ nanoclusters. The average bond length (Å) and angle bending (°) of CO_2_ molecule is depicted. Green, orange, black, and red spheres represent Co, Cu, C, and O atoms, respectively.

The most stable model of CO_2_ adsorption on the Co_13_ and Cu_13_ nanocluster is depicted in Figure 5a and Figure 5b, respectively. The results show that CO_2_ bends when it is adsorbed on the Co_13_ and Cu_13_ nanoclusters. Although the CO_2_ interaction on the Co_13_ nanocluster is consistent with the findings of other works [30], the interaction of CO_2_ and Cu_13_ differs from the one reported in previous studies, because it was reported that CO_2_ remained linear once adsorbed and only interacted with one Cu atom [30]. However, this distinct interaction might be attributed to the fact that they used nonicosahedral Cu_13_ nanoclusters.

To estimate the interaction between the CO_2_ molecule and the 3*d* transition-metal nanoclusters, the E_ads_ were calculated. In the case of the CO_2_ adsorbed on the Co_13_ nanocluster, the E_ads_ was −0.94 eV. This value is like those reported in the literature [30,49]. The E_ads_ calculated for CO_2_ on the Cu_13_ nanocluster was −0.18 eV, which agrees with the data reported in literature [30,49]. In addition, an average bond elongation of the CO_2_ was observed when it was adsorbed on the Co_13_ nanocluster. Regarding the bending angle, there was a pronounced curving with a reduction of 23.94% with respect to the isolated CO_2_ molecule. These parameters are comparable to those reported in previous works [30]. Concerning the average bond length and bending angle of the CO_2_ molecule adsorbed on the Cu_13_ nanocluster. As in the CO_2_ adsorbed on the Co_13_ nanocluster, an average bond elongation was observed. On the other hand, the bending angle underwent a considerable contraction of 28.34%. Nevertheless, these parameters do not concur with the values reported in earlier findings. In fact, Ocampo-Restrepo did not report this kind of modification in either the average bond length or bending angle of the CO_2_ molecule [30].

### 3.3. CO_2_ Adsorption on Co_13_ and Cu_13_ Nanoclusters Supported on PNG

**Figure 6:**
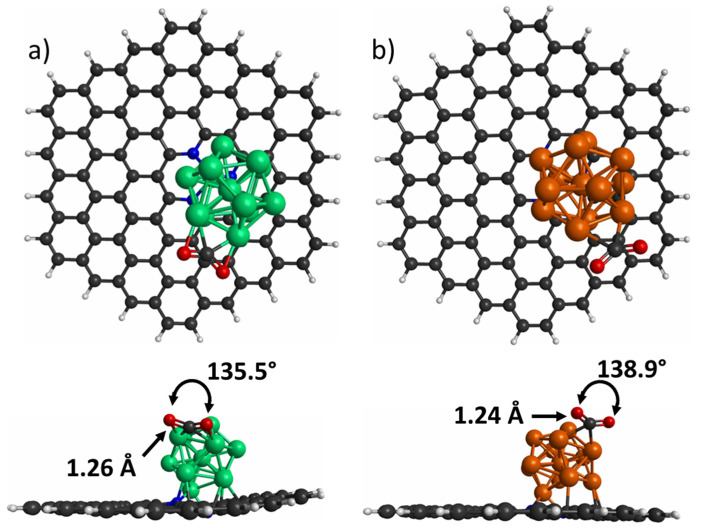
CO_2_ adsorption on (**a**) Co_13_/PNG and (**b**) Cu_13_/PNG. The bond length (Å) and angle bending (°) of CO_2_ molecule is depicted. Green, orange, black, white, blue, and red spheres represent Co, Cu, C, H, N, and O atoms, respectively.

To investigate the CO_2_ adsorption on Co_13_/PNG and Cu_13_/PNG composites, we analyzed various adsorption modes described in the preceding section. Again, the E_ads_, the average bond length, and the bending angle were determined to evaluate the CO_2_ adsorption. The most optimal adsorption mode for Co_13_/PNG and Cu_13_/PNG composites is depicted in Figure 6a and Figure 6b, respectively. For both composites, the CO_2_ preferred to be adsorbed in a bent way. Subsequently, the E_ads_ of CO_2_ on the Co_13_/PNG and Cu_13_/PNG composites was calculated. The results showed that the CO_2_ adsorbs more intensely on the Co_13_/PNG composite with a value of −0.92 eV, whereas the E_ads_ of CO_2_ on the Cu_13_/PNG composite was only −0.33 eV. As in the CO_2_ adsorbed on bare clusters, an average bond elongation of CO_2_ was observed when it was adsorbed on nanoclusters supported on PNG (Figure 6). Moreover, the CO_2_ bending angle underwent a considerable contraction when it was adsorbed on Co_13_ (24.72%) and Cu_13_ (22.83%) supported on PNG.

### 3.4. Bonding Analysis of the CO_2_ Interaction on Nanoclusters and Nanoclusters Supported on PNG

Table 2 shows the CO_2_ charge transference when is adsorbed on Co_13_, Cu_13_, Co_13_/PNG, and Cu_13_/PNG. The total charge of the CO_2_ molecule resulted in negative values for all systems, which indicates that the CO_2_ molecule gained a charge after the adsorption. For instance, the CO_2_ adsorption on the Co_13_ nanocluster enabled the CO_2_ to gain a charge of −0.74 *e*, indicating that the charge is transferred from the nanocluster to the CO_2_ molecule. It was reported that the transfer plays a significant role in the activation of the CO*_2_* molecule [32,49].

**Figure 7:**
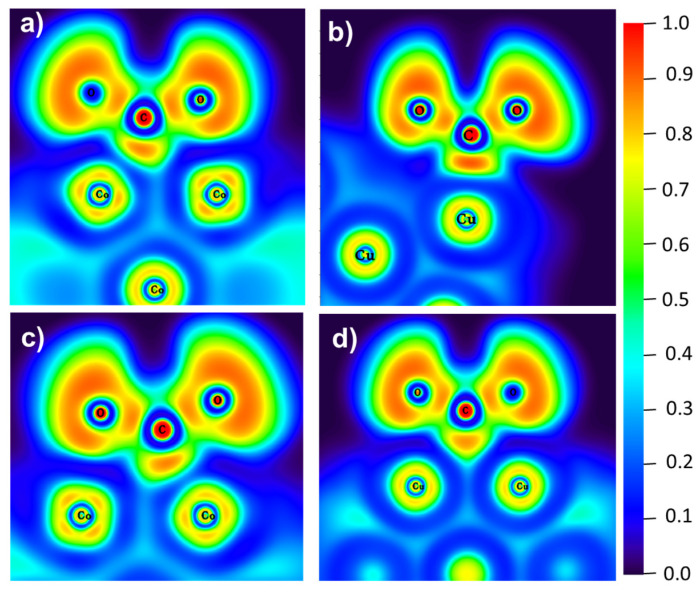
Electron localization function contours for: (**a**) CO_2_ adsorption on Co_13_, (**b**) CO_2_ adsorption on Cu_13_, (**c**) CO_2_ adsorption on CO_13_/PNG, and (**d**) CO_2_ adsorption on Cu_13_/PNG.

The electron localization function (ELF) was used to analyze the type of bond formed by the CO_2_ interaction on free nanoclusters and nanoclusters supported on PNG. ELF analysis is widely used to understand the nature of chemical bonds, which allows for differentiating covalent bonds and lone pairs (ELF = 1), as well as ionic bonds, hydrogen bonds, and van der Waals interactions (ELF = 0). Figure 7 shows the ELF plots for the CO_2_ adsorbed on nanoclusters and nanoclusters supported on PNG. As can be observed, the red region between the C atoms and the metal atom has ELF values of 0.8 and 0.9 for the C-Co (Figure 7a) and C-Cu bonds (Figure 7b), respectively. The high ELF value indicates a high charge transfer from the Cu_13_ to the CO_2_ molecules, in accordance with the total charge calculated (see Table 2). For the CO_2_ absorbed on the nanocluster/PNG, the ELF value is ~0.85 when the CO_2_ is absorbed on the Co_13_/PNG composite and ~0.8 when the CO_2_ is absorbed on the Cu_13_/PNG composite, suggesting a lower charge transfer from the metal cluster to the CO_2_ molecule (see Figure 7c,d). For the CO_2_ adsorption on Co_13_ and on Co_13_/PNG, a red region between the C and Co is observed, suggesting a bonding C-Co. Meanwhile, for CO_2_ on Cu_13_ a uniform red region is observed between C and a Cu. However, when CO_2_ is adsorbed on Cu_13_/PNG a semicircular region appears between C and two Cu atoms (see Figure 7d), suggesting that C is bonded with two Cu atoms.

## 4. Conclusions

The CO_2_ adsorption on bare 3*d* transition-metal nanoclusters and 3*d* transition-metal nanoclusters supported on the PNG was investigated using the ADFT. First, the interaction between Co_13_ and Cu_13_ nanoclusters with PNG was studied by spin densities, interaction energies, charge transfers, and HUMO-LUMO gaps. The results revealed that the PNG enhanced the stability and chemical reactivity of the 3*d* transition-metal nanoclusters. Subsequently, the CO_2_ was adsorbed on bare 3*d* transition-metal nanoclusters, and 3*d* transition-metal nanoclusters supported PNG. Numerous indicators such as bond elongation, angle bending, and charge transfer were used to characterize the CO_2_ interaction on these systems. In terms of bond elongation, bare nanoclusters and nanoclusters supported on PNG induced a CO_2_ bond elongation. In addition, the CO_2_ molecule experienced a bending angle when it was adsorbed on bare nanoclusters and nanoclusters supported on PNG. Charge transfer analysis revealed that the CO_2_ gained charge when it was adsorbed on 3*d* transition-metal nanoclusters and 3*d* transition-metal nanoclusters supported on PNG nanosheet. Although the nanoclusters and nanoclusters supported on PNG exhibited similar reactivity towards CO_2_, nanoclusters supported on PNG have the advantage of the support material providing excellent stability to the nanoclusters; therefore, they will not present agglomeration problems.

## Figures and Tables

**Table 1:** Charge transfers and HOMO-LOMO gaps for Co_13_, Cu_13_, Co_13_/PNG, and Cu_13_/PNG. The positive values indicate charge transfers from the nanoclusters to the PNG structure.

Systems	HOMO-LOMO Gap (eV)	Charge Transfer (*e*)
Co_13_	0.52	-
Cu_13_	0.67	-
Co_13_/PNG	0.26	+1.40
Cu_13_/PNG	0.21	+0.78

**Table 2:** Charge transfer from Co_13_, Cu_13_, Co_13_/PNG, and Cu_13_/PNG to CO_2_ molecule. The negative values indicate charge transfers from the nanoclusters and nanoclusters/PNG to the CO_2_ molecule.

Systems	Charge Transfer toward CO_2_ (*e*)
Co_13_	−0.74
Cu_13_	−0.78
Co_13_/PNG	−0.75
Cu_13_/PNG	−0.67

## Data Availability

Not applicable.
